# Effect of Community of Residence on Neurobehavioral Development in Infants and Young Children in a Flower-Growing Region of Ecuador

**DOI:** 10.1289/ehp.9261

**Published:** 2006-09-06

**Authors:** Alexis J. Handal, Betsy Lozoff, Jaime Breilh, Siobán D. Harlow

**Affiliations:** 1 Department of Epidemiology, University of Michigan School of Public Health, Ann Arbor, Michigan, USA; 2 Center for Human Growth and Development and Department of Pediatrics and Communicable Diseases, University of Michigan, Ann Arbor, Michigan, USA; 3 Health Research and Advisory Center (CEAS), Quito, Ecuador

**Keywords:** child development, Ecuador, flower industry, organophosphorus compounds, pesticides

## Abstract

**Objective:**

In this study we compared neurobehavioral development in Ecuadoran children living in two communities with high potential for exposure to organophosphate (OP) and carbamate pesticides to that of children living in a community with low potential for exposure.

**Methods:**

Women residing in the study communities who had a child 3–61 months of age completed a questionnaire about maternal and child health and sociodemographic characteristics. The Ages and Stages Questionnaire (ASQ) was administered to each child (*n* = 283). Growth measurements and a hemoglobin finger-prick blood test were obtained. We used multiple linear regressions to evaluate associations between community of residence and delayed development, adjusting for child health status and other characteristics of the home environment.

**Results:**

Children 3–23 months of age who resided in high-exposure communities scored lower on gross motor (*p* = 0.002), fine motor (*p* = 0.06), and socioindividual (*p*-value = 0.02) skills, compared with children in the low-exposure community. The effect of residence in a high-exposure community on gross motor skill development was greater for stunted children compared with non-stunted children (*p* = < 0.001) in the same age group of 3–23 months. Children 24–61 months of age residing in the high-exposure communities scored significantly lower on gross motor skills compared with children of similar ages residing in the low-exposure community (*p* = 0.06).

**Conclusions:**

Residence in communities with high potential for exposure to OP and carbamate pesticides was associated with poorer neurobehavioral development of the child even after controlling for major determinants of delayed development. Malnourished populations may be particularly vulnerable to neurobehavioral effects of pesticide exposure.

The adverse effects of environmental chemicals on neurologic development and function have been of interest for several decades. Previous research has focused on effects of exposure to polychlorinated biphenyls (PCBs) (Schantz et al. 2003; Seegal 1996), metals such as lead (Banks et al. 1997; Counter et al. 1998; Dietrich et al. 1987) and methylmercury (Gilbert and Grant-Webster 1995; Marsh et al. 1995), and organochlorine pesticides (Guillette et al. 1998). Although the organophosphate (OP) and carbamate classes of pesticides are in widespread use, their potential adverse impacts have received scant attention. These pesticides are less environmentally persistent than organochlorines, but animal evidence suggests that they are developmental neurotoxicants (Chanda and Pope 1996; Houeto et al. 1995; Miller 1982).

A few studies have reported potential adverse effects of OP and carbamate pesticide exposure on neurobehavioral development in young children. Young and colleagues (2005) found associations between prenatal exposure to OP pesticides and abnormal reflexes in neonates. Ruckart and colleagues (2004) conducted a study in Mississippi and Ohio examining the long-term neurobehavioral effects of exposure to the OP methyl parathion. Results of this study suggested possible problems with motor skills, attention, and short-term memory. Rohlman and colleagues (2005) found that children who resided in an agricultural community displayed poorer response speed and latency abilities compared with children from nonagricultural communities in the United States. Recently, Grandjean and colleagues (2006) reported an association between prenatal occupational exposure to OPs and neurotoxic damage among schoolchildren living in a flower-growing region of Ecuador.

The global use of pesticides has doubled every 10 years since 1945, and this trend is expected to continue in the following decades, with about half of the increase in pesticide use occurring in developing countries in the context of large-scale agricultural industries (World Health Organization 1990). In Ecuador, large-scale agricultural products dominate the export industry, and the cut-flower industry, which depends heavily on the use of pesticides, has become the country’s third most valuable export, behind oil and bananas. According to research conducted by the Centro de Estudios y Asesoría en Salud (CEAS) in Quito, Ecuador, the most commonly used classes of pesticides in this industry are OPs, carbamates, and dithiocarbamates (Breilh et al. 2005). Some examples of the pesticides used in this industry include mancozeb, methylbromide, captan, carbofuran, malathion, and diazinon. Few community-based studies of the effects of pesticide exposure on neurobehavioral development have been conducted in Ecuador (Grandjean et al. 2006).

This preliminary study was conducted under the auspices of the EcoSalud Project, a collaboration between CEAS and the International Development Research Center. In the present study we compare neurobehavioral development of infants and young children 3 to 61 months of age in communities that are dominated largely by cut-flower production and in more traditional rural communities.

## Materials and Methods

### Study population

The EcoSalud Project was launched in 2001 after local community leaders and members raised concerns about potential health problems among workers in the cut-flower industry and among community residents. The project investigates the impact of the Ecuadoran cut-flower industry in the Cayambe-Tabacundo region of Ecuador. As a component of the epidemiologic aspect of the EcoSalud project, this study focused on neurobehavioral development in infants and young children in the region.

The sample population for the EcoSalud project was drawn from four main study areas within the Cayambe-Tabacundo region, two northern sections and two southern sections with a total of 12 communities within the overall study area. The northern and southern areas were each divided into two distinct regions: One cluster of communities is located at a lower altitude in an area where the cut-flower industry is dominant; a second cluster of communities is located at a higher altitude and farther away from the flower plantations.

This study focused on three communities in the northern region. The communities were selected on the basis of exposure status and close ties between community leaders and the researchers at CEAS, allowing for greater accessibility to the community. A census was taken in each community to construct the sampling frame. All mothers who had been living in the community for at least 1 year and who had one or more children 3–61 months of age were eligible to participate. Up to three eligible children per mother were included. Informed consent was obtained from the mothers for their participation as well as that of their children. Consent forms were read to the mother, and consent was documented by the mother’s signature or fingerprint. In total, 219 eligible mothers (91.3% of total eligible) and 283 eligible children (91.0% of total eligible) participated in the study. Approval for this project was obtained from the institutional review board at the University of Michigan as well as from CEAS in Quito, Ecuador.

### Procedures

The Ages and Stages Questionnaire (ASQ), a developmental screening test, was directly administered to the child (Squires et al. 1999). Two trained testers assessed the participating children in each of the three communities. After administration of the ASQ, mothers were interviewed to obtain information on sociodemographic characteristics, maternal occupational history, maternal and child health characteristics, and the child’s socialization and exposure profiles. A finger-prick blood sample was obtained to assess the child’s hemoglobin levels (i.e., anemia status) using the HemoCue hemoglobin blood testing kit (HemoCue, Inc., Lake Forest, CA, USA). Height (centimeters), weight (kilograms), and head circumference (centimeters) of the child were measured, as were the height and weight of the mother. All survey instruments were pretested to ensure clarity and comprehensibility of the questions and pilot-tested to ensure agreement in assigned developmental scores between the two testers.

### High- and low-exposure communities

In this analysis, we considered community of residence as our main exposure variable. Communities A and B are lower-altitude communities located in proximity to the large, industrial flower farms and considered to have a higher potential for exposure to pesticides used in flower production. Households are more likely to be exposed environmentally and occupationally. Homes in these communities are located next to or in close proximity to the large cut-flower greenhouses, and preliminary findings by CEAS show that most young adults in these communities work in the flower industry (CEAS, unpublished data). These communities have less access to land to cultivate private crops, have less direct access to potable water, and depend mainly on food bought at the local markets. Women are likely to work outside the home and their children are likely to attend a local child care center. Flower workers do not bring home work clothes, but contamination of the local, open irrigation ditches and water systems is a potential source of exposure for children in these communities.

Community C is a higher-altitude community located far from the greenhouses with less likelihood of exposure to wind drifts and water contamination from the flower farms. Fewer community members, approximately 20%, work in the flower farms. This higher-altitude community relies mainly on local agriculture and crop production for food. They have direct access to potable water sources from mountain water runoff. Mothers more often work within the home and tend crops and cattle. Infants and young children typically stay home with their mother and older siblings.

### Neurobehavioral development assessment

The use of a parent-report screening test has been shown to be an effective and valid way to assess a child’s developmental progress (Glascoe 2000). The ASQ, a widely used screening instrument, is standardized for use in children 3 to 61 months of age and is composed of 19 age-specific questionnaires that cover five broad developmental dimensions: communication (vocalizing, listening, understanding), fine motor (hand, finger movements), gross motor (arm, leg, body movement), problem solving (learning, playing with toys), and personal–social (solitary social play, play with toys and other children) skills. Each domain is scored from 0 to 60 points, with 60 being a perfect score. A continuous score is calculated for each age-specific ASQ with scores summarized for each developmental domain. A cutoff for delayed development has been determined from a U.S. standardization sample, based on a score < 2 standard deviations of the standard score, for each given ASQ domain and age interval (Squire et al. 1999). The ASQ is available in several different languages including English, Spanish, French, and Korean.

Before administering the ASQ (Spanish version), we adapted the tool into the local vernacular. Contextually inappropriate questions were removed to prevent cultural and language bias (e.g., use of forks is not common practice in the region). Additionally, all references to the baby/child were changed to the Quichua term *guagua*. Testing was conducted using the home-visit procedure outlined in the ASQ manual, in which the tester attempts to elicit all ASQ behaviors directly from the child during the assessment. This procedure is slightly different than one that relies solely on parent report and is more appropriate in a setting where the parent may not be able to complete the questionnaire on her own, as was the case in our study population (Squire et al. 1999). Testers brought all materials required for direct assessment as listed in the ASQ manual. Mothers were encouraged to participate in the activities with their child throughout the session. Only when a particular activity could not be carried out or elicited at the time of the interview was a maternal report of the child’s behavior at home obtained.

### Covariates

Standardized *z*-scores for anthropometric measures of chronic malnutrition were calculated using the 1978 Centers for Disease Control (CDC)/World Health Organization growth reference curves, a normalized version of the 1977 National Center for Health Statistics growth reference curves (Dibley et al. 1987). Chronic malnutrition (stunting) was defined as the child’s height-for-age *z*-score 2 standard deviations below the median. We determined presence of anemia (yes/no) after taking into account the child’s age and the altitude of the community of residence (Table 1) (CDC 1989). Information on birth weight was obtained from the child’s vaccine record card. If the mother did not have this card, she was asked directly. Given the high frequency of missing information, low birth weight (LBW) was examined as a three-level variable (< 2,500 g, ≥ 2,500 g, or missing).

We assessed stimulation by two variables: attendance at the child care center (yes/no) and the type and frequency of stimulating activities the mother engaged in at home with each child in the preceding 3 days. For the latter, a set of six questions was adapted from a UNICEF (United Nations Children’s Fund) multicountry survey to assess home support for child development (UNICEF 2003). The six activities included reading, counting and/or drawing, looking at pictures (from any type of media), singing songs, going out of the house together, and playing together. A mother answered the questions based on her activities with the child in the past three days. To assess the home environment in which the child lives, mothers were also asked how they would rate their relationship with their husbands, or their families in the case of single mothers (good, calm, indifferent, or tense).

Socioeconomic characteristics included mother’s education level, father’s education level, mother’s ethnicity (indigenous, Mestizo/white), her predominant language preference (Quichua/Spanish mix, Spanish only), marital status (single, separated or widowed, or living as a free union or married), maternal age, monthly household income (US$0–150, $151–250, or > $250), and housing construction. Maternal education, categorized as none/partial elementary, completed primary school, or partial/completed high school, was used to assess education level and served as a proxy for literacy. Mother’s education and ability to read and write were correlated (*r* = 0.52, *p* = < 0.001; and *r* = 0.54, *p*-value < 0.001, respectively). Father’s education level was categorized similarly. Maternal age was examined as a continuous variable and also dichotomized at the median age (≤ 25 years, > 25 years).

Housing characteristics including roof composition, floor composition, wall composition, type of water used in home, bathroom type, and access to electricity were summarized into a housing scale, with possible scores ranging from 0 to 7. This housing scale was then categorized as poorer (≤ 3), mid-level (4–5), and better (6–7) housing construction based on distribution quartiles.

We also examined socioeconomic status by considering the social context of this particular region (Breilh J, unpublished data). A variable representing social position (social insertion) was based on the job of the principal economic provider of the family, because social position in this region depends highly on the job one holds. The categories for this variable were that the principal economic provider works in the flower industry, is a small business owner, or works in salaried manual labor. There is not a defined hierarchy among these categories, but working in the flower industry is valued and does provide a higher income.

## Statistical Analysis

Developmental delay within age-appropriate categories (3–23 months and 24–61 months) was analyzed separately for each developmental domain screened by the ASQ instrument. Given our limited sample size, we wanted to assess development within age-appropriate groups but were not able to create smaller age groupings (i.e., 1-year groupings). The two age groupings were chosen based on the distribution of the children’s ages in our population, consistency of results in preliminary analyses, and *a priori* planned future analyses that focus on prenatal exposure in the younger age group and environmental exposures in the older age group. We compared the distributions of the child’s health status, maternal characteristics, and the sociodemographic characteristics of the child’s family between the low- and high-exposure communities. Chi-square and *t*-test statistics were calculated.

Within the younger age category, two sets of sibling pairs were identified; among the older children, there were 18 sibling pairs. Comparisons between estimates from the correlated and uncorrelated regression models showed similar results. To eliminate any clustering in the sample, we randomly removed one sibling from each pair. The subsequent analyses of the data use this uncorrelated data set.

We assessed associations between the community of residence and each developmental domain. We constructed regression models to examine the effect of community of residence on child development, after controlling for health and sociodemographic characteristics. In a previous publication, we examined the health and sociodemographic factors associated with ASQ scores in this study population (Handal et al., in press). Because of the small sample size, only those variables found to be associated with each ASQ domain in that analysis were included in the regression model for a given domain. We constructed regression models with and without the LBW variable to examine possible differences in the estimates due to this variable. Potential interactions between the covariates and community of residence were also assessed. We calculated effect size (ES) to compare the magnitude of effect of community of residence on the developmental scores across the high- and low-exposure communities (Cohen 1998). The measure of ES, Cohen’s *d*, is calculated by taking the difference in the mean score of each exposure group divided by the standard deviation and is independent of sample size. Effect size is cautiously interpreted as small, *d* = 0.2; medium, *d* = 0.5; and large, *d* = 0.8. Given the relatively small sample size, we report both significant associations (*p* ≤ 0.05) and suggestive trends (*p* = 0.06–0.10). Data were entered into SPSS version 11.5 (SPSS Inc., Chicago, IL, USA) and were analyzed in SPSS and SAS version 8 (SAS Institute Inc., Cary, NC, USA). Nutritional data were analyzed in EpidInfo’s NutStat program software (CDC, Division of Public Health Surveillance and Informatics, Atlanta, GA, USA).

## Results

Overall, 154 children residing in the high-exposure communities (Communities A and B) and 129 children residing in the low-exposure community (Community C) were interviewed. There were 123 children 3–23 months of age and 160 children 24–61 months of age. After removing siblings, 121 children 3–23 months of age and 142 children 24–61 months of age were included in the analyses.

Table 2 shows the characteristics of participants by community of residence. Presence of anemia, LBW, stimulation at home, and attendance at child care all differed significantly across the communities. In the low-exposure community (Community C) compared with the high-exposure communities (A and B), more children presented with anemia (68.2% vs. 53.9%) and fewer attended child care (6.2% vs. 20.1%), whereas the high-exposure communities had more LBW children (17.5% vs. 3.1% in Community C). Although only about 63% of the entire sample reported a birth weight for the child, the percentage of missing LBW data was equally distributed between the high- and low-exposure communities.

In the low-exposure community, more mothers self-identified as indigenous (88.9%) and reported speaking some Quichua, the indigenous language of the region (16.3%), compared with the high-exposure communities (66.4%, *p* < 0.0001; and 7.1%, *p* = 0.02, respectively). A greater number of women in this community also reported having poor relations at home compared with the high-exposure communities (40.3% vs. 23.4%, *p* < 0.01). More families earned a monthly household income ≤ $150 in Community C (55%) compared with Communities A and B (40.8%). Better housing construction was reported in the high-exposure communities (39%) compared with the low-exposure community (19.4%) (*p* < 0.001). Finally, half of the principal economic providers for the family (as designated by the mother as the person principally responsible for the economic welfare of the family) in Communities A and B worked in the flower industry (50.7%) compared with 23% in Community C.

**Figure 1:**
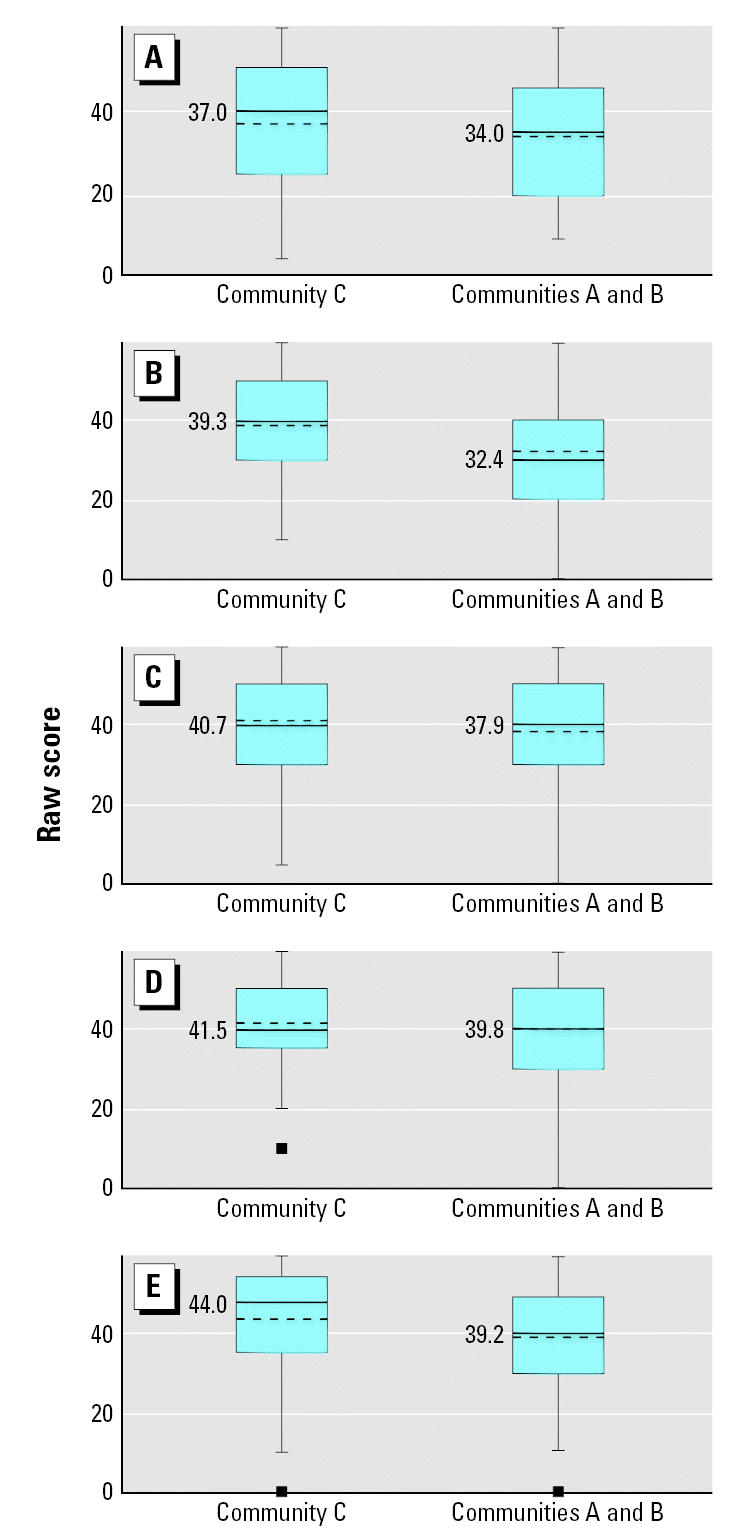
Unadjusted means of the five ASQ developmental domains by community of residence for children 3–23 months of age (*n* = 121). (*A*) Communication (*p* = 0.28), (*B*) Gross motor (*p* = 0.02), (*C*) Fine motor (*p* = 0.25), (*D*) Resolution of Problems (*p* = 0.49), and (*E*) Socioindividual (*p* = 0.05). The dashed line indicates the mean score for each community and the corresponding mean value is displayed to the left of the dashed line. The solid line indicates the median score for each community. Pairwise *t*-tests were used to assess the mean difference in ASQ score between community comparison groups.

**Figure 2:**
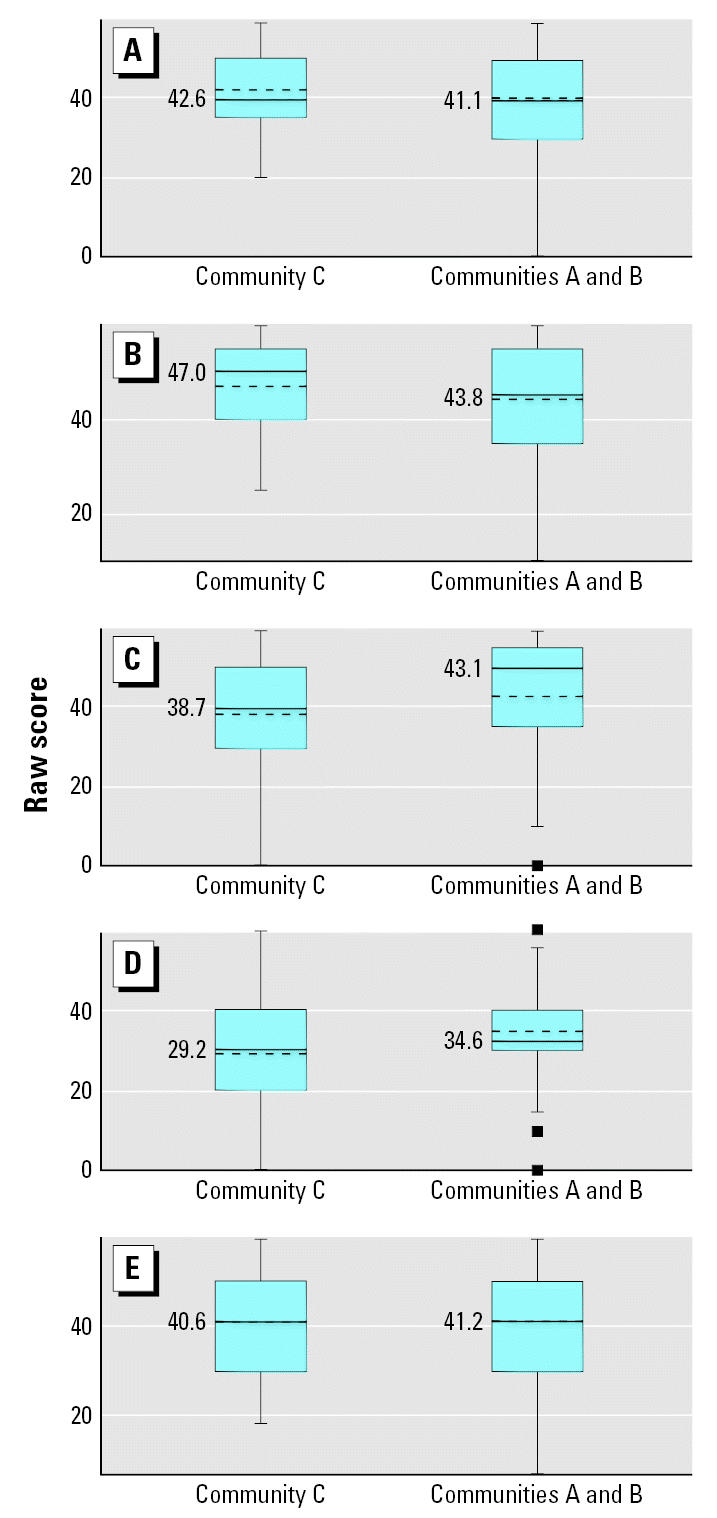
Unadjusted means of the five ASQ developmental domains by community of residence for children 23–61 months of age (*n* = 142). (*A*) Communication (*p* = 0.48), (*B*) Gross motor (*p* = 0.09), (*C*) Fine motor (*p* = 0.05), (*D*) Resolution of Problems (*p* = 0.01), and (*E*) Socioindividual (*p* = 0.74). The dashed line indicates the mean score for each community and the corresponding mean value is displayed to the left of the dashed line. The solid line indicates the median score for each community. Pairwise *t*-tests were used to assess the mean difference in ASQ score between community comparison groups.

**Figure 3:**
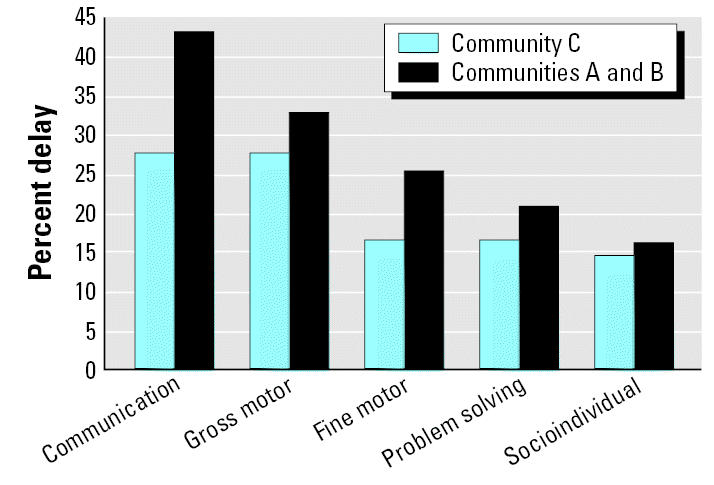
Percentage of children displaying developmental delay for the five ASQ developmental domains, stratified by community of residence for children 3–23 months of age (*n* = 123).

**Figure 4:**
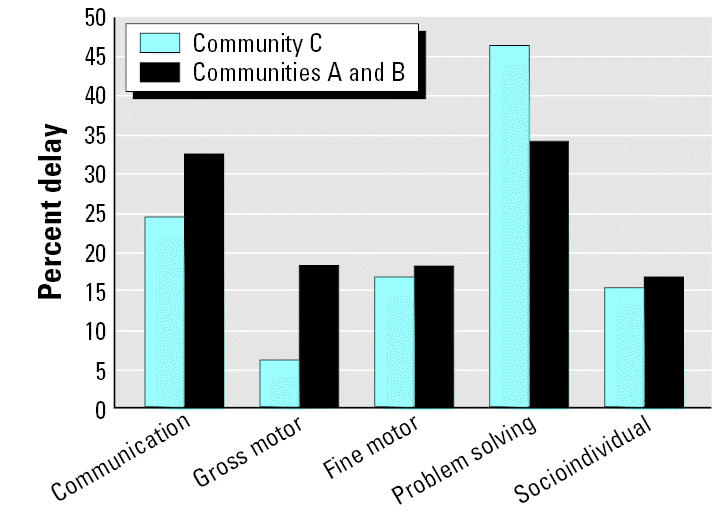
Percentage of children displaying developmental delay for the five ASQ developmental domains, stratified by community of residence for children 24–61 months of age (*n* = 160).

Figures 1 and 2 display the raw means of the five ASQ developmental domains by community of residence for the younger and older age categories, respectively. Among the younger children, scores were lower for all five developmental domains in Communities A and B compared with Community C (Figure 1), although only three of the five domains were statistically significant. In the older children, communication and gross motor scores were lower in Communities A and B compared with the low-exposure community (C). Fine motor, problem solving, and socioindividual scores in the older age group were higher in the high-exposure communities compared with Community C (Figure 2). Figures 3 and 4 display the percent delay for each of the five ASQ developmental domains by community of residence for the younger and older children, based on normative scores in a U.S. standardized population. A greater percent delay was observed in the high-exposure communities A and B across all five developmental domains for the younger age group (Figure 3). Among the older children, percent delay was greater in Communities A and B for communication, gross and fine motor, and socioindividual skills, based on a U.S. standardized population (Figure 4), even though the overall unadjusted means for fine motor and socioindividual skills were better than for those children residing in the low-exposure community.

Table 3 displays the results of the linear regression models for each of the developmental domains of the ASQ within each age group, adjusted for relevant health and sociodemographic differences between the high- and low-exposure communities. In the younger age group, community of residence remained significantly associated with gross and fine motor skills and socioindividual skills, after controlling for potentially confounding variables. Compared with children in Community C, children in the younger age group who resided in the high-exposure Communities A and B scored on average 8.8 points lower on the gross motor skill section of the ASQ (*p* = 0.002; ES: Cohen’s *d* = 0.4), 5.0 points lower on the fine motor skills *(p* = 0.06; ES: *d* = 0.2), and 5.8 points lower in the socioindividual skills (*p* = 0.02; ES: *d* = 0.3).

In the older age category, the only suggestive trend of association between community of residence and delayed development was in the gross motor skills domain. After controlling for potential confounders, older children residing in the high-exposure communities scored 3.8 points lower on their gross motor skills compared with older children residing in Community C (*p* = 0.06; ES: *d* = 0.2). Inclusion of LBW into the models did not substantively change these estimates.

Presence of stunting appeared to modify the results in each age group. Younger children who resided in the high-exposure communities and also suffered from chronic malnutrition (i.e., stunting) scored 17 points lower on their gross motor skills than did children in that same community who did not present with stunting and all children residing in the low-exposure community C (*p* < 0.001). The effect of community of residence on gross motor skill development in younger children was also greater for stunted children than for nonstunted children; however, because cell sizes were small, these results should be interpreted cautiously.

## Discussion

This study found that, in children 3 to 23 months of age, residence in the low-altitude communities dominated by cut-flower production with a high potential for exposure to pesticides was associated with developmental delay in all five developmental domains. In older children, 24 to 61 months of age, delays were present in two of five developmental domains. These results provide evidence that community of residence is associated with delayed neurobehavioral development of the child even after controlling for other predictors of delayed development such as low stimulation at home, presence of anemia, and stunting.

We observed poorer development in Communities A and B, especially in the younger age group, despite higher socioeconomic status, higher maternal education, more maternal stimulation, and fewer cases of anemia—all factors that should improve neurobehavioral development. We found greater frequencies of developmental delay across the five ASQ domains for children residing in these high-exposure communities compared with those children residing in the low-exposure community. These results warrant further investigation into the potential impact of pesticide exposure and other health and sociodemographic factors associated with presence of the industrial flower farms that may be contributing to delayed neurobehavioral development in these communities.

Research shows that chronic malnutrition (i.e., stunting) is associated with delayed neurobehavioral development (Delemarre-van de Waal 1993; Winick 1971), specifically with lowered gross and fine motor skills (Benefice et al. 1996; Cheung et al. 2001). In our study, a high percentage of stunting was observed. We found that children who resided in the high-exposure communities and who were stunted scored worse on gross motor skills than did the nonstunted children. That is, the effect of community exposure on gross motor skills was greater in the stunted children than in the nonstunted children. This malnourished sub-population may be particularly vulnerable to neurobehavioral effects of pesticide exposure.

Other factors such as attendance in child care and stimulation in the home may also modify the effect of community of residence on neurobehavioral development. Children who attended child care in Communities A and B scored significantly better in their fine motor skills. It may be that these children are exposed to more stimulating activities at the child care that aid in their fine motor development, compared with children who do not attend child care or who are more isolated and live in the rural sectors of Community C.

LBW is a strong predictor of delayed development (Breslau et al. 1996, 2004). Previous research has also suggested that exposure to pesticides, particularly organophosphates, may be associated with compromised fetal growth and gestational length (Eskenazi et al. 2004; Perera et al. 2003). In this sample, a larger percentage of mothers reporting LBW lived in the high-exposure communities. LBW may also be associated with work in or proximity to the cut-flower industry. However, because there was considerable nonresponse to questions about birth weight, we could not explore this potential association. Future studies in this region should consider including LBW as an additional outcome.

There are several limitations to this study. We focused on potential community-level differences in neurobehavioral development associated with proximity of the cut-flower industry to the community. We relied on an indirect exposure measurement (i.e., community of residence), which does not detail the specific potential pathways of pesticide exposure and may lead to exposure misclassification. Subsequent papers will address potential prenatal exposure to pesticides and childhood environmental exposures and their association with neurobehavioral development in these communities. We had information on the types of pesticides commonly used in the cut-flower industry, but data on the quantities and possible mixtures were not available. We were not able to obtain information on the type or quantity of pesticides used domestically, another potential source of measurement error. Future investigations should incorporate the use of bio-markers and environmental sampling.

There are limitations to using a general screening tool like the ASQ in this population. For most sections of the ASQ and especially for the fine and gross motor sections, children were directly tested by having them attempt various tasks such as crawling or walking and grasping blocks or toys. These activities were easier to test and score because they were observed directly. Other questions, however, depended on the mother’s recall of events at home. Specifically, the social skills section of the ASQ involved questions relating to how the child reacts to other children or siblings or how the child plays with his or her toys at home. Another limitation is the lack of standardized comparison data for the ASQ. In our analysis, we compared our results with a standardized U.S. population because information of a standardized Ecuadoran comparison population is not available. Validation of these developmental tools in this population and in similar Andean cultures is needed.

Another challenge in studying neurobehavioral development is that these processes can be subtle and alterations may be difficult to detect, especially if the exposure is low level and chronic. General screening tools such as the ASQ may not capture subtle delays that may be present. It is thus even more striking that these differences were observed. More precise measures of development are warranted to pursue the findings of this study. Neurobehavioral development is a dynamic process that is continuously changing as children get older. In this study, children were grouped into two age categories to account for some of this variability. Ideally, however, it would be best to group ages in 1-year categories or, with infants, in months. Despite these limitations, this survey is an important first step to understanding the patterns of child development in this population and raises important questions regarding the impact of the cut-flower industry on health in this region.

Knowledge of the effects of chronic exposure to pesticides in infants and young children, as may be the case in this region of Ecuador, is limited. This preliminary study suggests poorer neurobehavioral development in infants and children residing in high-exposure communities, especially in the development of gross motor skills. Our findings also suggest that there may be a double burden of pesticide exposure and poor nutrition on the neurobehavioral development in infants and young children in the developing world, but that child care and increased stimulation at home may attenuate these effects.

## Figures and Tables

**Table 1:** Cut-offs for anemia based on altitude and hemoglobin level (g/dL).

Community	Population	Age (years)	Hg (g/dL)
A^a^	Infants	< 2	< 12.3
	Girls/boys	2–5	< 12.5
B^b^	Infants	< 2	< 12.6
	Girls/boys	2–5	< 12.8
C^c^	Infants	< 2	< 13.0
	Girls/boys	2–5	< 13.2

a 8,530 ft (2,600 m) altitude.

b 9,515 ft (2,900 m) altitude.

c 10,433 ft (3,180 m) altitude.

**Table 2:** Characteristics of participants by community of residence (*n* = 283).

Characteristic	No.	Communities A and B (*n* = 154) No. (%)	Community C (*n* = 129) No. (%)	Chi-square (*p*-value)
Child’s age (months)				
3–24	123	68 (55.3)	55 (44.7)	0.07 (0.80)
24–16	160	86 (53.8)	74 (46.3)	
Child’s sex				
Male	150	83 (53.9)	67 (51.9)	0.12 (0.74)
Female	133	71 (46.1)	62 (48.1)	
Anemia in child				
No	112	71 (46.1)	41 (31.8)	6.02 (0.01)
Yes	171	83 (53.9)	88 (68.2)	
Stunting in child				
No	132	71 (46.1)	61 (47.3)	0.04 (0.84)
Yes	151	83 (53.9)	68 (52.7)	
LBW				
Missing data	104	60 (39.0)	44 (34.1)	18.79 (< 0.0001)
No (≥ 2,500 g)	148	67 (43.5)	81 (62.8)	
Yes (< 2,500 g)	31	27 (17.5)	4 (3.1)	
Child care attendance				
Yes	39	31 (20.1)	8 (6.2)	11.46 (0.0007)
No	244	123 (79.9)	121 (93.8)	
Mother’s age (years)				
≤ 25	143	83 (53.9)	60 (46.5)	1.53 (0.22)
> 25	140	71 (46.1)	69 (53.5)	
Ethnicity of mother				
Indigenous	213	101 (66.4)	112 (88.9)	19.37 (< 0.0001)
Mestizo/white	65	51 (33.6)	14 (11.1)	
Missing	5	2	3	
Language most used by mother				
Spanish/Quichua mix	32	11 (7.1)	21 (16.3)	5.84 (0.02)
Spanish	251	143 (92.9)	108 (83.7)	
Mother’s marital status				
Single	35	18 (11.7)	17 (13.2)	4.53 (0.21)
Separated/widowed	11	4 (2.6)	7 (5.4)	
Free union	72	46 (29.9)	26 (20.2)	
Married	165	86 (55.8)	79 (61.2)	
Mother’s education level				
None or partial elementary	70	40 (26.0)	30 (23.2)	2.02 (0.36)
Completed primary school	168	86 (55.8)	82 (63.6)	
Partial or completed high school	45	28 (18.2)	17 (13.2)	
Stimulation at home				
≥ 3 activities	163	109 (71.2)	54 (41.9)	24.8 (< 0.0001)
< 3 activities	119	44 (28.8)	75 (58.1)	
Missing	1	1		
Relations at home				
Good/calm	195	118 (76.6)	77 (59.7)	9.39 (0.0022)
Indifferent/tense/violent	88	36 (23.4)	52 (40.3)	
Monthly household income (US$)				
0–150	133	62 (40.8)	71 (55.0)	6.33 (0.04)
151–250	78	45 (29.6)	33 (25.6)	
> 250	70	45 (29.6)	25 (19.4)	
Missing	2	2		
Father’s education level				
None or partial elementary	42	20 (15.1)	22 (21.0)	1.52 (0.47)
Completed primary school	130	76 (57.6)	54 (51.4)	
Partial or completed high school	65	36 (27.3)	29 (27.6)	
Missing	46	22	24	
Housing construction				
Low	56	21 (13.6)	35 (27.1)	15.94 (0.0003)
Medium	142	73 (47.4)	69 (53.5)	
High	85	60 (39.0)	25 (19.4)	
Principal economic provider of family				
Salaried/subsalaried	100	65 (42.2)	35 (27.1)	66.09 (< 0.0001)
Own small business, military, retired	75	11 (7.1)	64 (49.6)	
Work in flowers	108	78 (50.7)	30 (23.3)	

**Table 3:** Adjusted regression models for five developmental domains for high-exposure compared with low-exposure communities.

	Age 3–23 months (*n* = 121)				Age 24–61 months (*n* = 142)			
ASQ	df	β (SE)	*p*-Value	Model adj *R*^2^	df	β (SE)	*p*-Value	Model adj *R*^2^
Communication	120	−3.66 (2.51)^a^	0.15	0.08	141	−0.06 (2.24)^b^	0.98	0.04
Gross motor	119	−8.80 (2.80)^c^	0.002	0.16	140	−3.83 (1.98)^d^	0.06	0.14
Fine motor	120	−5.00 (2.61)^e^	0.06	0.03	141	1.80 (2.41)^f^	0.46	0.05
Resolution of problems	120	−1.67 (2.42)^g^	0.49	0.02	141	3.69 (2.05)^h^	0.07	0.27
Socioindividual	120	−5.81 (2.36)^i^	0.02	0.17	140	−0.91 (2.21)^j^	0.68	0.02

Abbreviations: adj, adjusted; df, degrees of freedom.

a Adjusted for age of child (continuous), mother’s education.

b Adjusted for age of child (continuous), presence of anemia.

c Adjusted for age of child (continuous), presence of anemia, stunting, housing construction, monthly household income.

d Adjusted for age of child (continuous), stimulation at home, housing construction.

e Adjusted for presence of anemia, stimulation at home, housing construction.

f Adjusted for attendance at child care.

g Adjusted for age of child (continuous), mother’s education.

h Adjusted for age of child (continuous), mother’s education, relations at home.

i Adjusted for age of child (continuous), stimulation at home.

j Adjusted for stimulation at home.
